# Prevalence of right bundle branch block and its impact on recurrence after ablation in patients with atrial fibrillation

**DOI:** 10.3389/fcvm.2026.1790398

**Published:** 2026-04-13

**Authors:** Huimin Chu, Binhao Wang, Xiaojie Wang, Yiheng Yang, Xu Han, Xiaolei Yang, Yunlong Xia

**Affiliations:** 1 Department of Cardiology, The First Affiliated Hospital of Dalian Medical University, Dalian, Liaoning, China; 2 School of Medicine, Ningbo University, Ningbo, Zhejiang, China; 3 Arrhythmia Center, The First Affiliated Hospital of Ningbo University, Ningbo, Zhejiang, China

**Keywords:** atrial fibrillation, predictive factors, radiofrequency catheter ablation, recurrence, right bundle branch block

## Abstract

**Background:**

The prevalence of right bundle branch block (RBBB) among patients with atrial fibrillation (AF) and its prognostic value after radiofrequency catheter ablation (RFCA) remain unclear. This study aimed to investigate the prevalence of complete RBBB (CRBBB) and incomplete RBBB (IRBBB) and to evaluate their impact on recurrence after RFCA in patients with AF.

**Methods:**

A total of 949 consecutive AF patients who underwent *de novo* RFCA between 2018 and 2020 were retrospectively analyzed. Patients were categorized into non-RBBB, IRBBB, and CRBBB groups. Baseline clinical characteristics were compared among groups. Kaplan–Meier analysis and Cox proportional hazards regression models were used to assess the predictive value of RBBB for recurrence.

**Results:**

The prevalence of CRBBB and IRBBB among AF patients was 5.3% (*n* = 50) and 4.4% (*n* = 42), respectively, and both increased with aging (*p* < 0.001). During a median follow-up of 790 days (interquartile range: 495–1086 days), the recurrence rates in the non-RBBB, IRBBB, and CRBBB groups were 32.2%, 23.8%, and 56.0%, respectively. Log-rank analysis revealed significant differences in sinus rhythm maintenance among the three groups (*χ^2^* = 10.357, *p* = 0.006). After adjustment for confounding factors, multivariate Cox regression analysis demonstrated that CRBBB was an independent predictor of recurrence after RFCA (HR = 1.83; 95% CI: 1.04–3.23; *p* = 0.037), whereas IRBBB was not significantly associated with recurrence.

**Conclusion:**

The prevalence of RBBB is relatively high among AF patients undergoing RFCA and increases with aging. CRBBB may be an independent predictor of recurrence, whereas IRBBB is not associated with recurrence. The results should be proved by prospective multicenter investigation in the future.

## Introduction

Atrial fibrillation (AF) is one of the most common cardiac arrhythmias encountered in clinical practice (1). In recent years, radiofrequency catheter ablation (RFCA) has become a safe and effective therapeutic approach for AF (2, 3), with a reported success rate ranging from 60% to 90% (4–6). However, recurrence after ablation remains relatively common, and numerous studies have been conducted to identify potential predictors of postprocedural recurrence.

Right bundle branch block (RBBB) is a frequently observed conduction abnormality in clinical settings. Previous studies have demonstrated that RBBB serves as a risk factor for various cardiovascular diseases and is associated with adverse outcomes (7, 8). Recently, Zhang et al. (9) reported a significant association between complete right bundle branch block (CRBBB) and AF in hospitalized patients with cardiovascular diseases. Similarly, Khan et al. (10) found that the prevalence of AF was higher among patients with concomitant RBBB. However, limited evidence is available regarding the predictive value of RBBB for recurrence following radiofrequency ablation of AF. Therefore, the present study aimed to investigate the prevalence of RBBB in patients with AF and to evaluate its impact on recurrence after RFCA.

## Methods

### Study population

Between January 2018 and December 2020, patients with AF who underwent *de novo* RFCA were consecutively enrolled. The inclusion criteria were as follows: (1) patients aged ≥18 years; (2) had preprocedural electrocardiography (ECG) examination; (3) transesophageal echocardiography performed prior to ablation to exclude left atrial or left atrial appendage thrombus; (4) Patients willing to participate in the study and who provided written informed consent. The exclusion criteria included (1) absence of preprocedural ECG; (2) preprocedural ECG indicated other types of intraventricular conduction disturbances; (3) thrombus in the left atrium or left atrial appendage was detected during preprocedural TEE; (4) presence of thyrotoxicosis, heart failure with New York Heart Association class IV, severe hepatic or renal dysfunction, acute coronary syndrome, or acute cerebral infarction. CRBBB was defined as (1) QRS duration ≥120 ms, (2) rsr′, rsR′, rSR′, or rarely a qR in leads V1 or V2, the R′ or r′ deflection is usually wider than the initial R wave, (3) S wave of greater duration than R wave or >40 ms in leads I and V6, (4) normal R peak time in leads V5 and V6 but >50 ms in lead V1. Incomplete RBBB (IRBBB) was defined with the same QRS morphology criteria as CRBBB but with a QRS duration between 110 and 119 ms (11). The study protocol was reviewed and approved by the Ethics Committee of the First Affiliated Hospital of Dalian Medical University (No. YJ-KY-2021–123).

### RFCA procedure

Antiarrhythmic drugs were stopped at least five half-lives before RFCA, and ECG was further recorded. RFCA was performed under local anesthesia combined with intravenous analgesia. The right femoral vein was punctured, and a multipolar diagnostic catheter (MicroPort EP MedTech Co., Ltd., Shanghai, China) was advanced into the coronary sinus under fluoroscopic guidance. The right femoral vein was further punctured twice, and long sheaths (Sinnop Medical Technology Co., Ltd., Beijing, China) were inserted. Transseptal puncture was performed, followed by intravenous administration of heparin (100 IU/kg) through an infusion pump to maintain the activated clotting time between 300 and 350 s. Through the long sheath, an ablation catheter (SmartTouch, Biosense Webster, USA; or TactiCath™, Abbott, USA) and a mapping catheter (PentaRay, Biosense Webster, USA; or circular mapping catheter, MicroPort EP MedTech Co., Ltd., Shanghai, China) were introduced into the left atrium. A three-dimensional electroanatomic mapping system (CARTO3, Biosense Webster, USA; or Ensite NavX, St. Jude Medical, USA) was used to reconstruct the left atrial and pulmonary vein anatomy, followed by point-by-point circumferential pulmonary vein isolation (PVI). The radiofrequency energy was set at 35 W, and the saline irrigation flow rate was maintained at 17 mL/min. After successful PVI, voltage mapping of left atrium was performed. Additional linear ablation or ablation of complex fractionated atrial electrograms was performed if low voltage zones were detected. If a trigger arose from superior vena cava after bilateral PVI was detected, segmental isolation of superior vena cava was required. If AF persisted after ablation, electrical cardioversion was performed to restore sinus rhythm. After ablation, bidirectional conduction block across the ablation lines was confirmed after 30 min.

### Follow-up

RFCA was performed under local anesthesia. The first three months after ablation were defined as the blanking period, during which all patients received antiarrhythmic and anticoagulant therapy. Antiarrhythmic drugs were discontinued after blanking period. Twenty-four-hour Holter monitoring was performed at 3-, 6-, 9-, and 12-months post-procedure, and subsequently every 6 months thereafter. If patients experienced symptoms such as palpitations or chest discomfort, a 12-lead ECG or 24-hour Holter monitoring was performed immediately. Recurrence after RFCA was defined as the occurrence of atrial tachycardia, atrial flutter, or AF lasting longer than 30 s beyond the blanking period.

### Statistical analysis

Continuous variables were expressed as mean ± standard deviation (SD), and categorical variables were presented as counts and percentages. Independent-samples t-tests were used to compare continuous variables between two groups. For categorical variables, data were expressed as number (percentage), and comparisons between groups were performed using the chi-square test or Fisher's exact test when appropriate. Kaplan–Meier analysis was used to estimate the sinus rhythm maintenance rate in each group, and the log-rank test was applied to compare differences between groups. Cox proportional hazards regression analysis was conducted to evaluate the predictive value of RBBB for recurrence after RFCA, with results expressed as hazard ratios (HRs) and 95% confidence intervals (CIs). Sub-analysis using the Fine and Gray's competing risk model to account for death as a competing risk for recurrence. All statistical analyses were performed using SPSS Statistics version 19.0 (IBM, Armonk, NY, USA) and R version 4.5.1 (https://www.R-project.org). A *p* value < 0.05 was considered statistically significant.

## Results

### Prevalence of RBBB

Between January 2018 and December 2020, a total of 995 patients with AF underwent *de novo* RFCA in the First Affiliated Hospital of Dalian Medical University were included. Of these patients, six exhibited other types of intraventricular conduction disturbances on preprocedural ECG, and data for several critical parameters were missing in 40 cases. Finally, a total of 949 patients with AF was included in the study.

**Figure 1:**
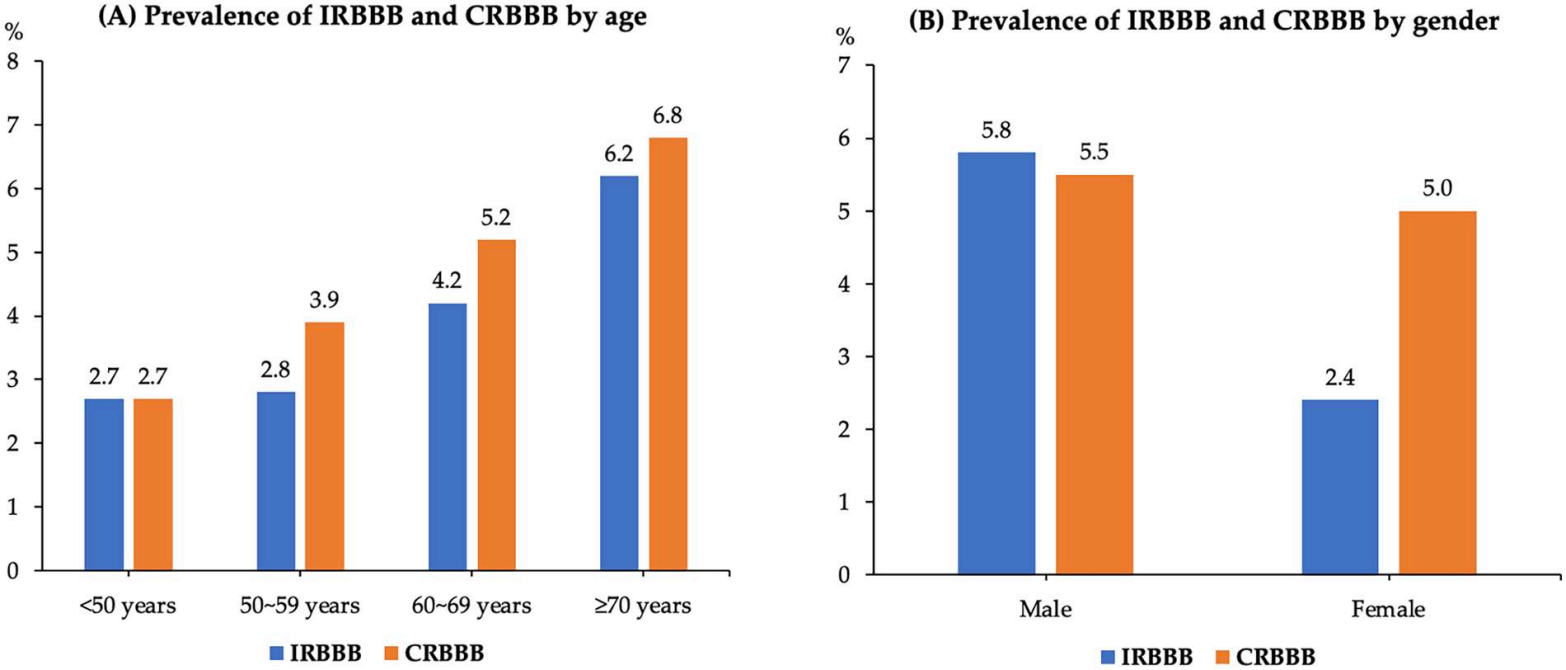
Prevalence of IRBBB and CRBBB in patients with AF undergoing RFCA by age **(A)** and gender **(B)**. IRBBB, incomplete right bundle branch block; CRBBB, complete right bundle branch block; AF, atrial fibrillation; RFCA, radiofrequency catheter ablation.

Among the enrolled patients, CRBBB and IRBBB were identified in 50 (5.3%) and 42 cases (4.4%), respectively. The prevalence of CRBBB among AF patients increased progressively with age, being 2.7%, 3.9%, 5.2%, and 6.8% in patients aged <50, 50–59, 60–69, and ≥70 years, respectively (*p* < 0.001). Similarly, the prevalence of IRBBB also increased with advancing age, being 2.7%, 2.8%, 4.2%, and 6.2% in the corresponding age groups (*p* < 0.001) (Figure 1). The prevalence of CRBBB was comparable between males and females (5.5% vs. 5.0%; *p* = 0.739), whereas that of IRBBB was significantly higher in males than in females (5.8% vs. 2.4%; *p* = 0.011) (Figure 1).

### Baseline characteristics

Compared with the non-RBBB group (*n* = 857), patients in the IRBBB group (*n* = 42) had a higher proportion of males, a lower prevalence of paroxysmal AF, a lower prevalence of diabetes mellitus, and a larger left atrial diameter. Compared with the non-RBBB group (*n* = 857), patients in the CRBBB group (*n* = 50) were older, had a lower prevalence of paroxysmal AF, a higher prevalence of heart failure, larger right and left atrial diameters, and a slightly lower left ventricular ejection fraction (LVEF) (Table 1).

**Table 1:** Patient characteristics.

Variables	Non-RBBB *n* = 857	IRBBB *n* = 42	CRBBB *n* = 50	*p* value*	*p* value**
Male (%)	503 (58.7)	33 (78.6)	31 (62.0)	0.011	0.739
Age (years)	64.2 ± 9.9	66.7 ± 9.9	67.4 ± 9.0	0.107	0.025
Type of Atrial fibrillation				0.035	0.050
Paroxysmal (%)	553 (64.5)	20 (47.6)	25 (50.0)		
Persistent (%)	304 (35.5)	22 (52.4)	25 (50.0)		
Hypertension (%)	398 (46.4)	17 (40.5)	26 (52.0)	0.426	0.421
Diabetes (%)	122 (14.2)	1 (2.4)	6 (12.0)	0.030	0.736
Coronary artery disease (%)	55 (6.4)	2 (4.8)	5 (10.0)	0.876	0.468
Heart failure (%)	191 (22.3)	10 (23.8)	18 (36.0)	0.908	0.026
CHA_2_DS_2_-VASc score				0.418	0.124
<2 (%)	407 (47.5)	17 (40.5)	18 (36.0)		
≥2 (%)	450 (52.5)	25 (59.5)	32 (64.0)		
QRS duration (ms)	86.8 ± 11.2	114.3 ± 2.7	126.5 ± 11.7	<0.001	<0.001
Right atrial diameter (mm)	33.1 ± 3.2	33.6 ± 3.0	35.8 ± 4.5	0.508	0.012
Left atrial diameter (mm)	39.4 ± 4.7	41.6 ± 6.5	40.9 ± 5.4	0.004	0.033
LVEF (%)	56.6 ± 6.0	56.2 ± 5.6	54.2 ± 7.5	0.697	0.007
Neutrophil to lymphocyte ratio	1.77 (1.34, 2.38)	2.06 (1.31, 2.63)	1.92 (1.53, 3.06)	0.318	0.029

RBBB, right bundle branch block; IRBBB, incomplete right bundle branch block; CRBBB, complete right bundle branch block; LVEF, left ventricular ejection fraction.

*p value: Comparison between the IRBBB group and the non-RBBB group.

**p value: Comparison between the CRBBB group and the non-RBBB group.

### Predictive value of RBBB for recurrence after RFCA of AF

**Figure 2:**
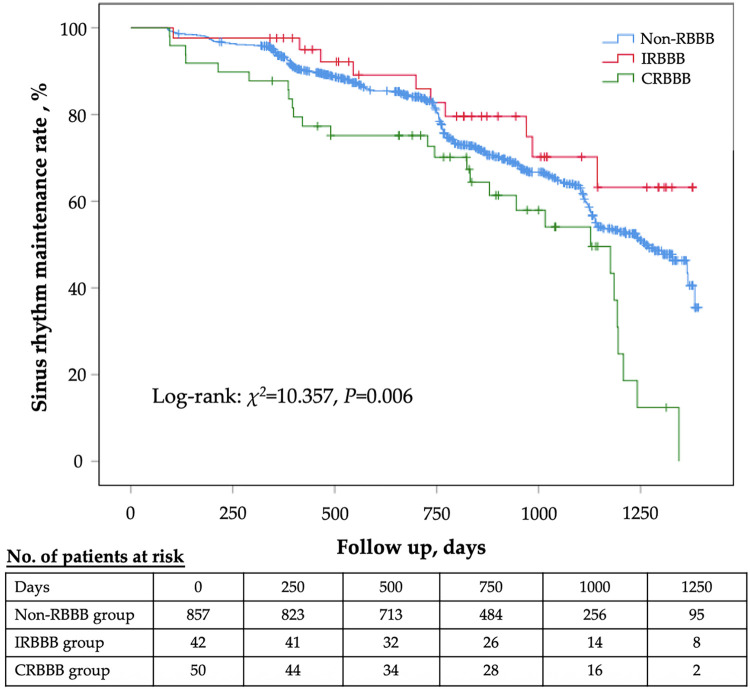
Kaplan–Meier curve of sinus rhythm maintenance rate for each group. RBBB, right bundle branch block; CRBBB, complete right bundle branch block; IRBBB, incomplete right bundle branch block.

After a median follow-up period of 790 (495, 1086) days, the number of recurrences in the non-RBBB, IRBBB, and CRBBB groups was 326 [326/857, 32.2%; 3–6 months, 16 (1.9%); 6–12 months, 38 (4.4%); >12 months, 272 (31.7%)], 10 [10/42, 23.8%; 3–6 months, 1 (2.4%); 6–12 months, 1 (2.4%); >12 months, 8 (19.0%)], and 28 [28/50, 56.0%; 3–6 months, 5 (10.0%); 6–12 months, 4 (8.0%); >12 months, 19 (38.0%)], respectively. Log-rank analysis revealed a statistically significant difference in sinus rhythm maintenance rates among the three groups (*χ^2^* = 10.357, *p* = 0.006) (Figure 2). In addition, 4 patients died before recurrence was detected. With the non-RBBB group as the reference, Cox regression analysis demonstrated that CRBBB was a significant predictor of AF recurrence after RFCA when adjusted for age and sex, whereas IRBBB showed no significant association with recurrence. CRBBB remained an independent risk factor for recurrence (HR = 1.83; 95% CI: 1.04–3.23; *p* = 0.037) after multivariate Cox regression analysis (including age, gender, AF type, coronary artery disease, hypertension, heart failure, diabetes, QRS duration, right atrial diameter, left atrial diameter, left ventricular ejection fraction, and neutrophil to lymphocyte ratio), whereas IRBBB was not associated with recurrence (Table 2). Sub-analysis using the Fine and Gray's competing risk model to account for death as a competing risk for recurrence showed a consistent result (Table 2).

**Table 2:** The value of RBBB in predicting recurrence after RFCA.

Groups	Number of recurrences (%)	Model 1^a^		Model 2^b^		Model 3^c^	
		HR (95% CI)	*p* value	HR (95% CI)	*p* value	HR (95% CI)	*p* value
Non-RBBB	326 (32.2)	Reference		Reference		Reference	
IRBBB	10 (23.8)	0.70 (0.37–1.31)	0.260	0.59 (0.29–1.23)	0.160	0.58 (0.28–1.20)	0.140
CRBBB	28 (56.0)	1.78 (1.19–2.64)	0.005	1.83 (1.04–3.23)	0.037	1.84 (1.06–3.20)	0.030

RBBB, right bundle branch block; IRBBB, incomplete right bundle branch block; CRBBB, complete right bundle branch block; RFCA, radiofrequency catheter ablation; HR, hazard ratio; CI, confidence interval.

a Adjusted for age and gender only.

b Adjusted for age, gender, AF type, coronary artery disease, hypertension, heart failure, diabetes, QRS duration, right atrial diameter, left atrial diameter, left ventricular ejection fraction, and neutrophil to lymphocyte ratio.

c Sub-analysis using the Fine and Gray's competing risk model to account for death as a competing risk for recurrence and adjustment for the same variables as the Model 2.

## Discussion

This study reported the prevalence of CRBBB (5.3%) and IRBBB (4.4%) among patients with AF in China. The findings demonstrated that the prevalence of both CRBBB and IRBBB increased with aging. Moreover, multivariate Cox regression analysis identified CRBBB as an independent predictor of recurrence after RFCA, whereas IRBBB was not associated with recurrence.

### Prevalence of RBBB in patients with AF

Data regarding the prevalence of RBBB among patients with AF are limited. Zhang et al. (9) enrolled 2,639 patients treated at the Cardiac Center of Henan Provincial People's Hospital, among whom 196 patients were diagnosed with AF (mean age: 63 ± 12 years; 59.7% male). CRBBB was detected in 9 patients (4.6%), which was comparable to that observed in our study; however, IRBBB prevalence was not analyzed in their study. Yano et al. (12) included 671 patients with AF who underwent RFCA in the Osaka Rosai AF Registry (Japan) (median age: 69 years; 63.6% male). Among them, 50 patients (7.5%) had CRBBB and 120 patients (17.9%) had IRBBB. The prevalence of both CRBBB and IRBBB in that study was higher than in our cohort, likely attributable to the older age of the study population. Overall, the prevalence of RBBB among AF patients is higher than that in the general community. This may be related to the higher prevalence of hypertension, diabetes mellitus, and heart failure among AF patients.

### Relationship between RBBB and AF

RBBB has often been regarded as a benign conduction abnormality previously. However, numerous studies have shown that RBBB is associated with adverse clinical outcomes. Bussink et al. (13) reported that CRBBB was associated with increased risks of both cardiovascular mortality and all-cause mortality. Patients with heart failure and concomitant RBBB have been shown to have a poorer prognosis (14, 15). In another study, approximately 10% of patients undergoing transcatheter aortic valve replacement had RBBB, which was identified as a predictor of adverse outcomes (16). Similarly, the presence of RBBB in patients with acute ST-segment elevation myocardial infarction was associated with a higher in-hospital mortality rate (17).

Investigations concerning recurrence after RFCA among patients with AF and RBBB remains scarce. Yano et al. (12) reported that after a median follow-up of 734 days, patients with CRBBB had a significantly higher risk of post-ablation recurrence (HR = 2.30; 95% CI: 1.00–5.33; *P* = 0.044). However, they did not investigate the relationship between IRBBB and recurrence after RFCA. Consistent with this finding, the present study also demonstrated that CRBBB was an independent predictor of AF recurrence after RFCA. In addition, IRBBB was not associated with recurrence.

The potential mechanisms of the increased risk of recurrence after RFCA in AF patients with CRBBB were as follows: (1) CRBBB causes asynchronous activation between early- and late-depolarized regions of the right ventricle, leading to ventricular mechanical dyssynchrony (18). In addition, CRBBB may result in abnormal atrioventricular coupling (19). These factors can contribute to right ventricular systolic dysfunction and tricuspid regurgitation, resulting in elevated right atrial pressure. Increased right atrial pressure is associated with right atrial enlargement, which has been closely linked to AF occurrence (20, 21). In the present study, patients with CRBBB exhibited larger right atrial diameters, which may support this hypothesis; (2) The prevalence of CRBBB increases with age, suggesting that it may serve as a marker of chronic degenerative changes in the cardiac conduction system, such as Purkinje network or myocardial fibrosis (22). Atrial fibrosis is a well-established substrate for AF initiation and maintenance. (3) The aforementioned structural and electrical remodeling may contribute to the formation of non–pulmonary vein triggers. A previous study of patients undergoing repeat ablation for AF recurrence found that those with CRBBB had a higher prevalence of non–pulmonary vein triggers (12). However, these mechanisms are hypothesized and should be proved by further investigations.

### Clinical implications

Significant procedural costs, x-ray exposure and potentially serious complications may accompany RFCA in AF patients. Recurrence after ablation is still the major concern in patients with AF. Therefore, better selection of patients is clinical significance. Several scoring systems were recently proposed to predict the risk of AF recurrence after the ablation (23, 24). ECG parameters were non-invasive, easily accessible, and reproducible. Various ECG parameters, including bundle branch blocks, have been associated with AF recurrences after ablation (25). The MB-LATER clinical score (male, bundle brunch block, left atrium ≥47 mm, type of AF, and early recurrent AF) was derived to predict late AF recurrence after RFCA (26), and further validated in a Chinese cohort (27). We also demonstrated that CRBBB may be an independent predictor of AF recurrence. In the present study, patients with CRBBB were older and had a higher prevalence of heart failure, larger atrial diameters and lower LVEF, which suggesting more advanced structural heart disease. In older patients with structural heart disease, CRBBB might reflect not only disturbance in the conduction system but also could be associated with atrial remodeling or ventricular dysfunction (28). Whether CRBBB is an independent causal factor of AF recurrence or a marker of advanced cardiac remodeling is difficult to determine based on current published evidence. However, we believe the results of the current investigation may provide reference to select potential patients to receive ablation in clinical setting. Preprocedural evaluation should be more comprehensive in patients with AF and CRBBB, especially accompanied by factors associated with structural remodeling.

### Limitations

This study has several limitations. First, it was a single-center retrospective study including only patients with AF who underwent RFCA; thus, the results require validation by large-scale, multicenter prospective studies. Second, the number of patients with RBBB was relatively small, which may have limited the statistical power; nonetheless, we believed that our findings provided meaningful clinical insights for electrophysiologists. Third, voltage mapping of the right atrium was not routinely performed during RFCA in this cohort, which may have led to an underestimation of non–pulmonary vein triggers. Fourth, the data regarding pulmonary disease was not available for most patients due to the retrospective design. Given the well-established association between pulmonary disease, RBBB, and AF recurrence, omission of these variables may be a potential source of confounding. Finally, AF recurrence detection relied on 24-hour Holter monitoring and symptom-driven ECGs, some asymptomatic episodes may have been missed.

## Conclusions

The prevalence of RBBB is relatively high among patients with AF undergoing RFCA and increases with age. CRBBB may be an independent predictor of recurrence, whereas IRBBB is not associated with recurrence. However, the results should be proved by prospective multicenter investigation in the future.

## Data Availability

The original contributions presented in the study are included in the article/Supplementary Material, further inquiries can be directed to the corresponding author.

## References

[1] McManus DD Rienstra M Benjamin EJ. An update on the prognosis of patients with atrial fibrillation. Circulation. (2012) 126:e143–6. 10.1161/CIRCULATIONAHA.112.129759 22949543 PMC3678907

[2] Joglar JA Chung MK Armbruster AL Benjamin EJ Chyou JY Cronin EM 2023 ACC/AHA/ACCP/HRS guideline for the diagnosis and management of atrial fibrillation. J Am Coll Cardiol. (2024) 83:109–279. 10.1016/j.jacc.2023.08.017 38043043 PMC11104284

[3] Van Gelder IC Rienstra M Bunting KV Casado-Arroyo R Caso V Crijns HJGM 2024 ESC guidelines for the management of atrial fibrillation developed in collaboration with the European Association for Cardio-Thoracic Surgery (EACTS). Eur Heart J. (2024) 45:3314–414. 10.1093/eurheartj/ehae176 39210723

[4] Zhang R Chu H Liu S Yang B Han B Xiao X Catheter ablation of atrial fibrillation using FireMagic TrueForce ablation catheter: the TRUEFORCE trial. Pacing Clin Electrophysiol. (2023) 46:986–93. 10.1111/pace.14751 37334721

[5] Urbanek L Bordignon S Schaack D Chen S Tohoku S Efe TH Pulsed field versus cryoballoon pulmonary vein isolation for atrial fibrillation: efficacy, safety, and long-term follow-up in a 400-patient cohort. Circ Arrhythm Electrophysiol. (2023) 16:389–98. 10.1161/CIRCEP.123.011920 37254781

[6] Ukita K Egami Y Kawamura A Nakamura H Matsuhiro Y Yasumoto K Clinical impact of very early recurrence of atrial fibrillation after radiofrequency catheter ablation. J Cardiol. (2021) 78:571–6. 10.1016/j.jjcc.2021.08.004 34426045

[7] Figueroa-Triana JF Mora-Pabón G Quitian-Moreno J Álvarez-Gaviria M Idrovo C Cabrera JS Acute myocardial infarction with right bundle branch block at presentation: prevalence and mortality. J Electrocardiol. (2021) 66:38–42. 10.1016/j.jelectrocard.2021.02.009 33770645

[8] Galcerá-Jornet E Consuegra-Sánchez L Galcerá-Tomás J Melgarejo-Moreno A Gimeno-Blanes JR Jaulent-Huertas L Association between new-onset right bundle branch block and primary or secondary ventricular fibrillation in ST-segment elevation myocardial infarction. Eur Heart J Acute Cardiovasc Care. (2021) 10:918–25. 10.1093/ehjacc/zuab026 33993235

[9] Zhang F-T Liu X-J Zhao D-Q Wu J-T Zhang L-M Hu J Association between complete right bundle branch block and atrial fibrillation development. Ann Noninvasive Electrocardiol. (2022) 27:e12966. 10.1111/anec.12966 35567783 PMC9296786

[10] Khan MZ Patel K Zarak MS Gupta A Hussian I Patel K Association between atrial fibrillation and bundle branch block. J Arrhythm. (2021) 37:949–55. 10.1002/joa3.12556 34386121 PMC8339096

[11] Kusumoto FM Schoenfeld MH Barrett C Edgerton JR Ellenbogen KA Gold MR 2018 ACC/AHA/HRS guideline on the evaluation and management of patients with bradycardia and cardiac conduction delay: a report of the American College of Cardiology/American Heart Association Task Force on Clinical Practice Guidelines and the Heart Rhythm Society. Circulation. (2019) 140:e382–482. 10.1161/CIR.0000000000000628 30586772

[12] Yano M Egami Y Ukita K Kawamura A Nakamura H Matsuhiro Y Impact of baseline right bundle branch block on outcomes after pulmonary vein isolation in patients with atrial fibrillation. Am J Cardiol. (2021) 144:60–6. 10.1016/j.amjcard.2020.12.051 33385350

[13] Bussink BE Holst AG Jespersen L Deckers JW Jensen GB Prescott E. Right bundle branch block: prevalence, risk factors, and outcome in the general population: results from the Copenhagen City Heart Study. Eur Heart J. (2013) 34:138–46. 10.1093/eurheartj/ehs291 22947613

[14] Aguiló O Castells X Miró Ò Mueller C Chioncel O Trullàs JC. The prognostic significance of bundle branch block in acute heart failure: a systematic review and meta-analysis. Clin Res Cardiol. (2023) 112:1020–43. 10.1007/s00392-022-02105-z 36116092

[15] Barsheshet A Goldenberg I Garty M Gottlieb A Sandach A Laish-Farkash A Relation of bundle branch block to long-term (four-year) mortality in hospitalized patients with systolic heart failure. Am J Cardiol. (2011) 107:540–4. 10.1016/j.amjcard.2010.10.007 21184999

[16] Auffret V Webb JG Eltchaninoff H Muñoz-García AJ Himbert D Tamburino C Clinical impact of baseline right bundle branch block in patients undergoing transcatheter aortic valve replacement. JACC Cardiovasc Interv. (2017) 10:1564–74. 10.1016/j.jcin.2017.05.030 28734885

[17] Juárez-Herrera U Jerjes Sánchez C González-Pacheco H Martínez-Sánchez C. In-hospital outcome in patients with ST elevation myocardial infarction and right bundle branch block. A sub-study from RENASICA II, a national multicenter registry. Arch Cardiol Mex. (2010) 80:154–8. 21147580

[18] Solodky A Zafrir N. Electrical and mechanical dyssynchrony in patients with right bundle branch block. J Nucl Cardiol. (2020) 27:631–3. 10.1007/s12350-018-1460-z 30298370

[19] Miller BE Rajsheker S López-Candales A. Right bundle branch block and electromechanical coupling of the right ventricle: an echocardiographic study. Heart Views. (2015) 16:137–43. 10.4103/1995-705X.172197 26900418 PMC4738494

[20] Nattel S Burstein B Dobrev D. Atrial remodeling and atrial fibrillation: mechanisms and implications. Circ Arrhythm Electrophysiol. (2008) 1:62–73. 10.1161/CIRCEP.107.754564 19808395

[21] Akutsu Y Kaneko K Kodama Y Suyama J Li H-L Hamazaki Y Association between left and right atrial remodeling with atrial fibrillation recurrence after pulmonary vein catheter ablation in patients with paroxysmal atrial fibrillation: a pilot study. Circ Cardiovasc Imaging. (2011) 4:524–31. 10.1161/CIRCIMAGING.110.962761 21778328

[22] Koshiyama M Tamaki K Ohsawa M. Age-specific incidence rates of atrial fibrillation and risk factors for the future development of atrial fibrillation in the Japanese general population. J Cardiol. (2021) 77:88–92. 10.1016/j.jjcc.2020.07.022 32800634

[23] Kornej J Hindricks G Shoemaker MB Husser D Arya A Sommer P The APPLE score: a novel and simple score for the prediction of rhythm outcomes after catheter ablation of atrial fibrillation. Clin Res Cardiol. (2015) 104:871–6. 10.1007/s00392-015-0856-x 25876528 PMC4726453

[24] Canpolat U Aytemir K Yorgun H Şahiner L Kaya EB Oto A. A proposal for a new scoring system in the prediction of catheter ablation outcomes: promising results from the Turkish Cryoablation Registry. Int J Cardiol. (2013) 169:201–6. 10.1016/j.ijcard.2013.08.097 24063932

[25] Karanikola AE Tzortzi M Kordalis A Doundoulakis I Antoniou C-K Laina A Clinical, electrocardiographic and echocardiographic predictors of atrial fibrillation recurrence after pulmonary vein isolation. J Clin Med. (2025) 14:809. 10.3390/jcm14030809 39941478 PMC11818469

[26] Mujović N Marinković M Marković N Shantsila A Lip GYH Potpara TS. Prediction of very late arrhythmia recurrence after radiofrequency catheter ablation of atrial fibrillation: the MB-LATER clinical score. Sci Rep. (2017) 7:40828. 10.1038/srep40828 28106147 PMC5247745

[27] Deng H Shantsila A Xue Y Potpara TS Bai Y Zhan X Using the MB-LATER score for predicting arrhythmia outcome after catheter ablation for atrial fibrillation: the Guangzhou atrial fibrillation project. Int J Clin Pract. (2018) 72:e13247. 10.1111/ijcp.13247 30144238

[28] Lewinter C Torp-Pedersen C Cleland JG Køber L. Right and left bundle branch block as predictors of long-term mortality following myocardial infarction. Eur J Heart Fail. (2011) 13:1349–54. 10.1093/eurjhf/hfr130 22027083

